# Automatic brain extraction and brain tissues segmentation on multi-contrast animal MRI

**DOI:** 10.1038/s41598-023-33289-7

**Published:** 2023-04-19

**Authors:** Jamil Nour Eddin, Hugo Dorez, Valentina Curcio

**Affiliations:** HawkCell, Marcy-l’Étoile, 69280 France

**Keywords:** Medical imaging, Nervous system, Imaging, Computational neuroscience, Image processing, Software

## Abstract

For many neuroscience applications, brain extraction in MRI images is the first pre-processing step of a quantification pipeline. Once the brain is extracted, further post-processing calculations become faster, more specific and easier to implement and interpret. It is the case, for example, of functional MRI brain studies, or relaxation time mappings and brain tissues classifications to characterise brain pathologies. Existing brain extraction tools are mostly adapted to work on the human anatomy, this gives poor results when applied to animal brain images. We have developed an atlas-based Veterinary Images Brain Extraction (VIBE) algorithm that encompasses a pre-processing step to adapt the atlas to the patient’s image, and a subsequent registration step. We show that the brain extraction is achieved with excellent results in terms of Dice and Jaccard metrics. The algorithm is automatic, with no need to adapt the parameters in a broad range of situations: we successfully tested multiple MRI contrasts (T1-weighted, T2-weighted, T2-weighted FLAIR), all the acquisition planes (sagittal, dorsal, transverse), different animal species (dogs and cats) and canine cranial conformations (brachycephalic, mesocephalic, dolichocephalic). VIBE can be successfully extended to other animal species, provided that an atlas for that specific species exists. We show also how brain extraction, as a preliminary step, can help to segment brain tissues with a *K*-Means clustering algorithm.

## Introduction

Brain extraction, also known as skull stripping, is a preliminary image post-processing technique that is fundamental for multiple applications in neuroscience and quantitative image analysis for clinical and research purposes. To perform quantitative analysis on the brain, a segmentation of the brain parenchyma is needed: functional Magnetic Resonance Imaging (fMRI), for example, is a technique that highlights activated regions in the brain, when the subject is stimulated with a specific stimulus pattern. To ensure that the considered activation signals are only those located in the brain, the analysis needs a preliminary skull stripping step. Skull stripping also enhances performances in the operation of inter-subjects brain normalisation, used to draw comparisons in fMRI results among different subjects^1^. Other examples are the calculations for quantitative MRI mappings applied to brain lesions characterisation, such as T1 and T2 relaxation times or quantitative susceptibility maps^2,3^. The brain extraction operation selects the brain as the only region of interest, leading to faster and more specific analysis, focused only on the brain itself. However, automatic brain extraction tools for the animal brain are still limited, and mostly dedicated to non-domestic animals such as rodents, macaque and marmoset^4–6^. We believe that a brain extraction tool that can be extended to multiple animal species, including domestic animals such as dogs and cats, can push the development of new quantitative neuroimaging tools to be integrated in veterinary research, clinical and pre-clinical practice.

One of the most successful brain extraction technique for humans is the atlas-based segmentation^7^. A brain atlas is elastically registered to the full anatomical image, and used to extract the brain parenchyma from surrounding non-brain tissues. Many popular automatic brain extraction tools include atlases in their process, such as 3dSkullStrip, part of the AFNI suite^8^, the Hybrid Watershed Algorithm^9^, combining watershed for initialisation and atlas registration, or the Skull Stripping function part of the Insight Toolkit (ITK), a popular library for medical image processing^10^. Since these tools use human atlases, they can not be adapted to animals, due to the variability of their anatomy depending on the species. Other popular tools, even if not explicitly based on human atlases, make use of hyperparameters tuned on the human skull conformation. It is the case of the Brain Extraction Tool (BET)^11^, available as part of the FMRIB Software Library (FSL), and of FreeSurfer^12^, that makes use of Bayesian priors. The Brain Surface Extraction (BSE) tool integrated in the BrainSuite^13,14^ is based on edge detection and morphological operations, such as erosion. The animal skulls, such as dogs’ and cats’, have a larger amount of tissues surrounding the brain, and the boundary between the brain and the skull is not always well contrasted. This makes it hard to separate non-brain tissues based only on intensity and morphological operations. The problem arises especially in the rostral portion of the brain (towards the nasal cavity and the buccal cavity), in the ventral portion of the brain (towards the nasopharynx), and laterally (towards the masticatory muscles). For these reasons, an automatic brain extraction algorithm based on animal morphology that can be used in veterinary clinical and research routines is needed. For it to be adapted to different animal morphologies, it needs to be based on atlases of a specific animal species. Nowadays, animal digital atlases are being created, that can be integrated in image processing pipelines. They are available for the dog^15–20^, the cat^21,22^, the domestic pig^23^, the sheep^24–26^, the horse^27^, the baboon^28,29^, the macaque^30,31^, the marmoset^32,33^ and the rat brain^34^.

We have developed VIBE (Veterinary Images Brain Extraction): an atlas-based brain extraction algorithm adapted to the animal’s anatomy. We are showing the application and robustness of VIBE in particular on domestic dogs and cats, but the principle can be applied to every animal species, provided that an MRI atlas exists. We are also showing that an atlas built on a specific MRI contrast can be successfully used for brain extraction of MRI volumes of multiple contrasts, resolutions and acquisition orientations. We quantitatively assess the quality of the brain extraction problem over a cohort of cats and dogs of different races and brain conformations. As an application, we also show how brain extraction as a preliminary step can simplify the brain tissues segmentation task, and leads to precise separation of the cerebrospinal fluid, white and gray matter.

## Methods

### Study population

All the animals chosen to be part of this retrospective study were patients of the veterinary clinic VetAgro Sup (Marcy-l’Étoile, France), who were prescribed an MRI examination, performed by HawkCell (Marcy-l’Étoile, France). All the images were anonymized to assure the patients’ confidentiality. All procedures were approved by the Comité d’éthique de VetAgro Sup ethics committee, stating that non-interventional studies, based only on the use of retrospective data, are subject to a non-opposition rule. We state that the manuscript follows the reporting recommendations in the ARRIVE guidelines^35^.

The canine cohort consists of 30 dogs of different breeds and cranial conformations, with no sign of brain abnormalities. We selected 10 brachycepahlic, 10 mesocephalic and 10 dolichocephalic dogs (see Table  1 for detailed information). We assigned the cranial conformation based on the brain length rather than the breed, following the criteria described by Milne et al.^16^ As for the feline cohort, it consists of 10 cats of different breeds, with no sign of brain abnormalities (see Table 2 for detailed information).

**Table 1:** Dogs study population by race, sex (F: Female, M: Male), age and cranial conformation.

Subject	Dog’s breed	Sex	Age (years)	Cranial conformation
1	American Staffordshire Terrier	F	10	Brachycephalic
2	Brittany	F	4	Brachycephalic
3	Brittany	F	13	Brachycephalic
4	Chihuahua	M	9	Brachycephalic
5	Eurasier	M	9	Brachycephalic
6	French Bulldog	F	6	Brachycephalic
7	French Bulldog	F	8	Brachycephalic
8	Jack Russel Terrier	M	7	Brachycephalic
9	Mixed breed	M	9	Brachycephalic
10	Yorkshire Terrier	M	14	Brachycephalic
11	American Staffordshire Terrier	M	5	Mesocephalic
12	Australian cattle dog	F	1	Mesocephalic
13	Australian Shepherd	M	2	Mesocephalic
14	Border collie	M	5	Mesocephalic
15	Cane corso	F	3	Mesocephalic
16	German shepherd	M	13	Mesocephalic
17	Golden retriever	F	12	Mesocephalic
18	Golden retriever	F	13	Mesocephalic
19	Husky	M	7	Mesocephalic
20	West Highland white terrier	M	9	Mesocephalic
21	Belgian Malinois	M	6	Dolichocephalic
22	Greater Swiss mountain dog	M	2	Dolichocephalic
23	Griffon Bleu de Gascogne	M	9	Dolichocephalic
24	Husky	M	3	Dolichocephalic
25	Husky	M	6	Dolichocephalic
26	Labrador	F	18	Dolichocephalic
27	Labrador	M	8	Dolichocephalic
28	Labrador	M	13	Dolichocephalic
29	Mixed breed	M	9	Dolichocephalic
30	Mixed breed	M	10	Dolichocephalic

**Table 2:** Cats study population by race, sex (F: Female, M: Male) and age.

Subject	Cat’s breed	Sex	Age (years)
1	Chartreux	M	16
2	European shorthair	F	1
3	European shorthair	F	3
4	European shorthair	F	12
5	European shorthair	F	21
6	European shorthair	M	3
7	European shorthair	M	6
8	European shorthair	M	12
9	European shorthair	M	12
10	European shorthair	M	14

### Imaging protocol

During the MRI scans, the animals were kept under general anesthesia, performed by veterinary anesthesiologists, following the European College of Veterinary Anaesthesia and Analgesia (ECVAA) guidelines. The animals were sedated with intravenous dexmedetomidine (1 $\upmu \text {g}/\text {kg}$) and induced to general anesthesia with propofol (2 mg/kg). Diazepam (0.2 mg/kg) was added in case the total anesthesia was not reached. The animals were then intubated and kept anesthetised with oxygen and inhalant isoflurane (1% to 2%) during the imaging procedure.

All the images were acquired with a 1.5T GE Signa Explorer MRI System. For each subject a 3D T1-weighted brain volume sequence (BRAVO; GE Healthcare), a T2-weighted Fast Relaxation Fast Spin Echo sequence (FRFSE) and T2-weighted FLuid-Attenuated Inversion Recovery (FLAIR) were acquired. The BRAVO sequence has been acquired along either the dorsal or the sagittal plane, with the following acquisition parameters: flip angle: 12°, inversion time: 450 ms, echo time: ranging between 3 ms and 3.46 ms, repetition time: 8 ms, bandwidth: ranging between 81.3 Hz/px and 122.07 Hz/px, isotropic in-plane pixel resolution ranging between 0.68 × 0.68 $\text {mm}^{2}$ and 0.81 × 0.81 $\text {mm}^{2}$, slice thickness ranging between 0.8 $\text {mm}$ and 1 $\text {mm}$ for dorsal acquisitions and between 1.2 $\text {mm}$ and 1.4 $\text {mm}$ for sagittal acquisitions. The T2 FRFSE sequence has been acquired along the sagittal plane, with the following acquisition parameters: flip angle: 160°, echo time: ranging from 98 ms and 105 ms, repetition time: ranging between 2847 ms and 4771 ms, bandwidth: 122.07 Hz/px, in-plane pixel resolution ranging between ${0.48 \times 0.62}$ $\text {mm}^{2}$ and ${0.55 \times 0.71}$ $\text {mm}^{2}$, slice thickness ranging between 2.2 $\text {mm}$ and 3.3 $\text {mm}$. The T2 FLAIR sequence has been acquired along the transverse plane, with the following acquisition parameters: flip angle: 160°, inversion time: ranging between 1824 ms and 2258 ms, echo time: ranging between 116.9 ms and 123.6 ms, repetition time: either 5000 ms or 7000 ms, bandwidth: ranging between 108.5 Hz/px and 122.1 Hz/px, in-plane pixel resolution ranging between ${0.46 \times 0.62}$ $\text {mm}^{2}$ and ${0.6 \times 0.8}$ $\text {mm}^{2}$, slice thickness ranging between 2.2 $\text {mm}$ and 3.8 $\text {mm}$.

### Brain extraction algorithm

**Figure 1:**
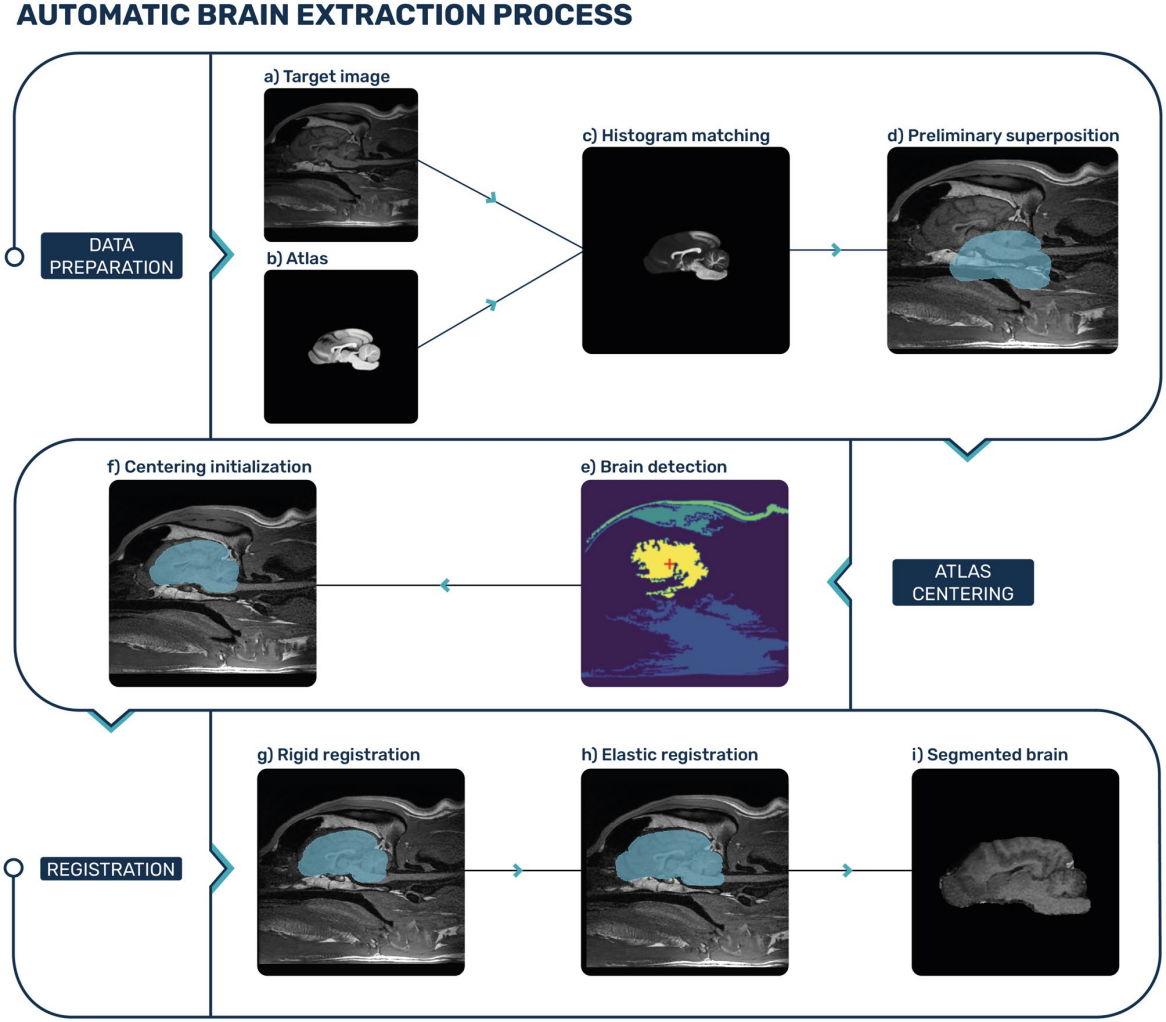
Scheme of the brain extraction algorithm workflow, example on a canine subject. (**a**) Target image of a MRI brain acquisition, in which we want to extract the brain. (**b**) Atlas chosen for the segmentation process. In this paper we used the atlas from Johnson et al.^20^. (**c**) Histogram matching between the atlas and the target image’s grayscale. (**d**) Preliminary superposition of the atlas over the target image. (**e**) Detection of the brain region using Felzenszwalb segmentation method: the target image is segmented into regions to locate the brain’s center. (**f**) Translation of the atlas center towards the target image’s brain center, to align both brains’ centers and ease the rigid registration step. (**g**) Rigid registration of the atlas for optimal atlas-to-target superposition. (**h**) Elastic registration of the atlas to fully adapt the atlas to the target’s brain and overcome any anatomical variability. (**i**) Completed brain extraction after the registration steps.

The VIBE algorithm uses an animal brain atlas to perform a fully automatic skull stripping on a brain MRI scan. The full workflow is illustrated in Fig. 1. For the canine population, we used the publicly available T1-weighted canine atlas published by Johnson et al.^20^. It is a high-resolution probabilistic atlas: the presence of mixed-breed subjects makes it more prone to adapt to a variety of canine breeds. For the feline population, a T1-weighted atlas of the same author was chosen^22^. The algorithm was written in Python, using especially the Python wrapping interface of the Insight ToolKit (ITK)^36^ and scikit-image^37^ as image processing libraries.

#### Data preparation

First, the target image (Fig. 1a) DICOM metadata were transposed to the atlas (Fig. 1b): resolution, size, physical position and orientation of the atlas are set to be the same as those of the target image. Resampling with linear interpolation was performed on the atlas to match the same voxel resolution and matrix size of the target image. The needed rotations and translations are performed to match the target image physical position and orientation, regardless of the native acquisition plane (sagittal, dorsal or transverse). For best performance of the registration steps, based on the voxels intensities, a histogram matching^38^ of the atlas intensity range to the subject’s image intensity range is performed (Fig. 1c).

#### Atlas centering

After resampling, the atlas and the patient’s brain are not necessarily centered (Fig. 1d): while the atlas is at the center of the field of view, the patient’s brain could be shifted. The registration process is harder if not properly initialised with a preliminary centering (Supplementary Fig. 1a). We are in the context of a model-to-image registration, a type of registration where the model image (the atlas) has only the brain portion in common with the patient’s image, while all the surrounding tissues are missing. The brain is thus the only portion of the target image that the atlas will be able to rely on. To superpose the atlas to the target image’s brain, we aim at locating the patient’s brain center. We used the Felzenszwalb algorithm^39^ (Fig. 1e) on the central slice of the acquired MRI volume to locate the brain. The scale of the expected regions to segment can be set as hyperparameter, which makes this graph-based segmentation algorithm ideal for the detection of objects of a roughly known size, such as the brain. The reference scale chosen to define the hyperparameters was the resampled atlas surface ($S_{atlas}$), calculated as the number of non-background pixels in the central 2D slice of the resampled atlas. The hyperparameters thus scale with the resolution of the target image, and the segmentation outcome does not depend on it. The scale of observation used is $k=200\, S_{atlas}$. A smoothing Gaussian filter with $\sigma = 0.5$ is applied to make Felzenswalb algorithm less dependent on details and noise. The region corresponding to the brain is selected as the one whose centroid is closest to a reference point, assigned to each anatomical plane (sagittal, dorsal and transverse), and representing the average brain’s position. The atlas is finally translated to match the position of the detected brain in the target image (Fig. 1f).

#### Rigid registration

To precisely superpose the atlas (used as moving image) over the brain of the subject (used as fixed image), a rigid transformation consisting of a 3D translation and a 3D rotation was performed (Fig. 1g). A gradient descent was used as optimizer for the registration (step size = 1, number of iterations = 50, convergence minimum value = $10^{-6}$ within a convergence window of size 10), and a B-spline interpolator to interpolate the moving image pixel intensities at non-grid positions. Mattes mutual information was chosen as similarity metric as it is well-established for inter-subject image registration^40,41^. The number of histogram bins was set to 50. To reduce computational time and help the registration procedure to focus on the brain region, we applied a mask to the fixed target image. The mask is chosen to correspond to the atlas brain mask, dilated with a 3D ball structuring element of radius 10 pixels.

#### Elastic registration

The elastic registration adapts the atlas shape to the exact brain morphology, highly variable between subjects (Fig. 1h). This is done by applying a local non-linear transformation to the image regions to fit the moving image to the fixed image. We used a free-form deformation technique guided by a B-spline transform. A grid with uniformly distributed control points is overlaid to the moving image and locally deformed to minimize the similarity metric. The grid points are set to be separated by 8 pixels for the 3D BRAVO sequence (for computation speed purposes), and by one pixel for the 2D T2 FRFSE and FLAIR sequences. Mattes mutual information was chosen as a similarity metric, and a quasi-newton Limited-memory Broyden–Fletcher–Goldfarb–Shanno optimizer for bound optimization (L-BFGS-B)^42^. L-BFGS-B is a good choice for elastic registration procedures since it works well with non-linear optimization problems and a high number of variables. The gradient convergence tolerance was set to $10^{-5}$, and the number of iterations to 15. A dilated binary mask of the atlas was also used to limit the target image’s virtual sampling points in order to make the elastic registration faster and more accurate (Supplementary Fig. 1b). For the BRAVO sequence a 3D ball structuring element of radius 10 pixels was used, while for the T2 FRFSE and FLAIR sequences a 3D ball structuring element of radius 1 pixel. Once the elastic registration is done, we created a binary mask of the registered atlas, and then this mask was multiplied by the target MRI image to obtain the final segmented brain (Fig. 1i).

### Comparison with existing algorithms

We compared VIBE to three existing and commonly used brain extraction tools: BET (the Brain Extraction Tool from FSL, version 2)^11^, AFNI’s 3dSkullStrip (3DSS)^8^ and 3D Pulse-Coupled Neural Networks (3D-PCNN)^4^. To select the optimal parameters, we randomly chose a dog and a cat from the respective cohorts. For each tool and each sequence, different parameters were tested. The parameters giving the highest Dice Similarity Coefficient result where chosen as the optimal ones. The optimal parameters were then extended to the whole dogs’ and cats’ cohorts. Detailed optimal parameters used for each tool are listed below.

BET algorithm: for the dogs cohort, a 0.8 fractional estimation threshold and the “BET2 Robust brain center estimation” mode were selected for all the three sequences (T1 BRAVO, T2 FRFSE, T2 FLAIR); for the cats cohort, a 0.8 fractional estimation threshold and the “BET2 Robust brain center estimation” mode were selected for T1 BRAVO and T2 FLAIR sequences, while a 0.5 fractional estimation threshold and the “standard brain extraction using BET2” mode were selected for T2 FRFSE images; the other parameters were kept to default for best performance.

3DSS algorithm: for the dogs cohort, an initial sphere radius of 80mm was used for T1 BRAVO and T2 FRFSE images, while an initial sphere radius of 45mm was used for the T2 FLAIR images; for the cats cohort, an initial sphere radius of 100mm was used for T1 BRAVO, T2 FRFSE and T2 FLAIR images. The other parameters were kept to default for best performance.

3D-PCNN algorithm: for the dogs cohort the brain size range used was [80,000-110,000] $\text {mm}^3$, the morphological structure’s radius was set to 23 for T2 FRFSE and 13 for T2 FLAIR; for the cats cohort the brain size range used was [25,000–37,000] $\text {mm}^3$, the morphological structure’s radius was set to 15 for T2 FRFSE and 11 for T2 FLAIR. The zoom was set to 1 for all the sequences and cohorts; no optimal parameters enabling us to perform brain extraction were found for the T1 BRAVO, in both cats and dogs. The Matlab code available on the authors’ website (https://sites.google.com/site/chuanglab/software/3d-pcnn) was used.

BET and 3DSS were run on a MacBook Pro (OS: macOS Monterey, CPU: 2GHz Intel Core i5 4 cores, memory: 16Gb, GPU: Intel Iris Plus Graphics 1536Mb), while 3D-PCNN was run on a workstation (OS: Windows 10 Professionnal, CPU: 2.10GHz Intel(R) Xeon(R) Silver 4216, RAM: 96Go, GPU: Nvidia Quadro GV100).

## Results

### Validation

To validate our brain segmentation algorithm, we compared the results with a manually segmented mask, considered as the ground truth. We used two similarity metrics to evaluate the output of our algorithm: the Dice Similarity Coefficient (DSC)^43^ and the Jaccard index^44^. The Dice similarity coefficient measures the overlap between a binary mask generated as the segmentation result and the manual segmentation binary mask using the following formula:

1 $$\begin{aligned} DSC = \dfrac{2|A \cap M|}{|A| + |M|}, \end{aligned}$$

with *A* being the output mask from the automatic segmentation algorithm, and *M* being the manually segmented mask. The DSC ranges from 0 to 1, with 1 being the best similarity score. The Jaccard index calculates the intersection of the two binary masks divided by their union:

2 $$\begin{aligned} J = \dfrac{|A \cap M|}{|A \cup M|}. \end{aligned}$$

Jaccard index also ranges from 0 to 1, and is expected to give a lower value than Dice similarity coefficient. For completeness, we also calculated the sensitivity and specificity. Sensitivity ranges from 0 to 1, with a value of 1 indicating that all the brain pixels have been included in the segmentation, and is calculated as follows:

3 $$\begin{aligned} Sensitivity = \dfrac{|A \cap M|}{|M|}. \end{aligned}$$

Specificity ranges as well between 0 and 1, with a value of 1 indicating that no background pixels were misclassified as part of the brain, and is calculated as follows:

4 $$\begin{aligned} Specificity = \dfrac{|V - M \cup A|}{|V - M|}, \end{aligned}$$

where *V* is the total volume of the image. It has to be noted that the Specificity values will tend to be very close to 1, since the pixels occupied by the brain mask are of the order of 5% of the total pixels in the image. It is thus to be used for comparisons among different conditions and not to determine absolute quality. While DSC and Jaccard index asses the overall quality of the segmentations, Sensitivity and Specificity, taken together, highlight the tendency of an automatic segmentation to over- or underestimate the brain region.

For each subject of our cohort, a manual segmentation has been accurately made using the 3D Slicer software^45^ by an experienced user, trained by veterinary specialists. In order to assess the quality of manual segmentations, two veterinary experts were asked to independently perform a manual segmentation of two different canine MR images. The veterinary segmentations were compared with those performed by the experienced user, giving a Dice score of 0.98 and a Jaccard index of 0.97. We thus consider that the experienced user’s segmentations are highly similar to those performed by veterinary experts.

### Brain extraction

We tested the VIBE algorithm taking into account multiple variables that can be encountered in veterinary MRI: we applied it to different animals (dogs and cats), different canine cranial conformations (brachycephalic, mesocephalic, dolichocephalic), different MRI sequences (T1 3D BRAVO, T2 FRFSE, T2 FLAIR) and different acquisition planes (sagittal, dorsal, transverse). We used the validation process described above, and compared the results to three other common brain extraction tools: BET, 3DSS and 3D-PCNN.

#### Tests on different MRI sequences

**Figure 2:**
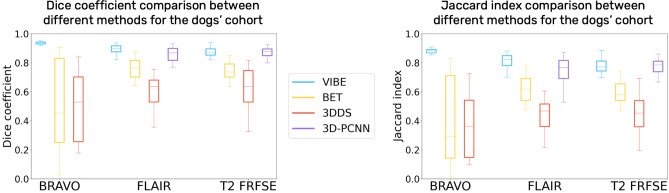
Dice similarity coefficient and Jaccard index Boxplot for the dogs cohort, comparing VIBE, BET, 3DSS and 3D-PCNN for three different sequences: T1 BRAVO, T2 FLAIR and T2 FRFSE.

**Figure 3:**
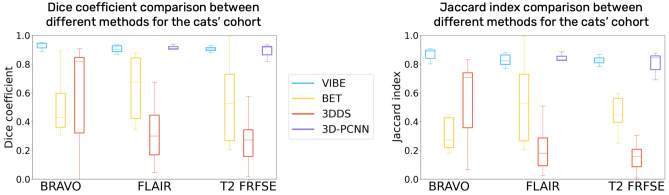
Dice similarity coefficient and Jaccard index Boxplot for the cats cohort, comparing VIBE, BET, 3DSS and 3D-PCNN for three different sequences: T1 BRAVO, T2 FLAIR and T2 FRFSE.

First, we tested our algorithm on 3D T1 BRAVO acquisitions. We expected to obtain the best results from this sequence, since the chosen atlas is built on images acquired using a 3D T1 BRAVO sequence as well, although on a 3T MRI machine. The histogram matching and resampling of the atlas did not require heavy modifications in terms of intensity and resolution to match the target BRAVO images, leading indeed to optimal results for both dogs and cats. The results of Dice similarity coefficient and Jaccard index are reported as average values and standard deviation over the cohort (Table 3 for the dogs cohort, Table 4 for the cats). They are represented as boxplots in Fig. 2 for the dogs, and Fig. 3 for the cats, to show the dispersion and skewness of the results.

**Table 3:** Dogs cohort results.

Dogs	T1 BRAVO		T2 FLAIR		T2 FRFSE	
	Dice	Jaccard	Dice	Jaccard	Dice	Jaccard
VIBE	$0.93 \pm 0.02$	$0.87 \pm 0.03$	$0.89 \pm 0.03$	$0.80 \pm 0.06$	$0.87 \pm 0.03$	$0.77 \pm 0.05$
BET	$0.5 \pm 0.3$	$0.4 \pm 0.3$	$0.75 \pm 0.08$	$0.60 \pm 0.09$	$0.74 \pm 0.06$	$0.59 \pm 0.07$
3DSS	$0.5 \pm 0.2$	$0.4 \pm 0.2$	$0.6 \pm 0.2$	$0.4 \pm 0.1$	$0.6 \pm 0.2$	$0.5 \pm 0.1$
3D-PCNN	/	/	$0.84 \pm 0.08$	$0.7 \pm 0.1$	$0.86 \pm 0.05$	$0.76 \pm 0.08$

Comparison of the mean and standard deviation results of both the Dice similarity coefficient and Jaccard index for three different MRI contrasts (T1 BRAVO, T2 FLAIR and T2 FRFSE) and different algorithms (VIBE, BET, 3DDS and 3D-PCNN).

**Table 4:** Cats cohort results.

Cats	T1 BRAVO		T2 FLAIR		T2 FRFSE	
	Dice	Jaccard	Dice	Jaccard	Dice	Jaccard
VIBE	$0.92 \pm 0.02$	$0.86 \pm 0.04$	$0.90 \pm 0.02$	$0.82 \pm 0.04$	$0.89 \pm 0.02$	$0.81 \pm 0.04$
BET	$0.5 \pm 0.2$	$0.4 \pm 0.2$	$0.6 \pm 0.2$	$0.4 \pm 0.2$	$0.6 \pm 0.1$	$0.45 \pm 0.11$
3DSS	$0.7 \pm 0.3$	$0.6 \pm 0.3$	$0.3 \pm 0.2$	$0.2 \pm 0.2$	$0.3 \pm 0.2$	$0.2 \pm 0.1$
3D-PCNN	/	/	$0.90 \pm 0.03$	$0.82 \pm 0.05$	$0.89 \pm 0.04$	$0.80 \pm 0.06$

Comparison of the mean and standard deviation results of both the Dice similarity coefficient and Jaccard index for three different MRI contrasts (T1 BRAVO, T2 FLAIR and T2 FRFSE) and different algorithms (VIBE, BET, 3DDS and 3D-PCNN).

We then tested the capability of VIBE to adapt to contrasts and resolutions different from the atlas, in order to prove its interest in a wide range of MRI acquisitions conditions. We applied it to transverse two-dimensional T2-weighted FLAIR images, and to sagittal T2-weighted FRFSE images. In both cases, the Dice similarity coefficient and the Jaccard index are high (see Table 3 for dogs, and Table 4 for cats). Different contrasts only slightly affect the quality of the registration outcome. A slightly lower quality in the similarity metrics with respect to the T1 BRAVO is due to the under-sampling of the three-dimensional atlas acquisition to a two-dimensional image, which might have lower precision due to partial volume effects.

**Figure 4:**
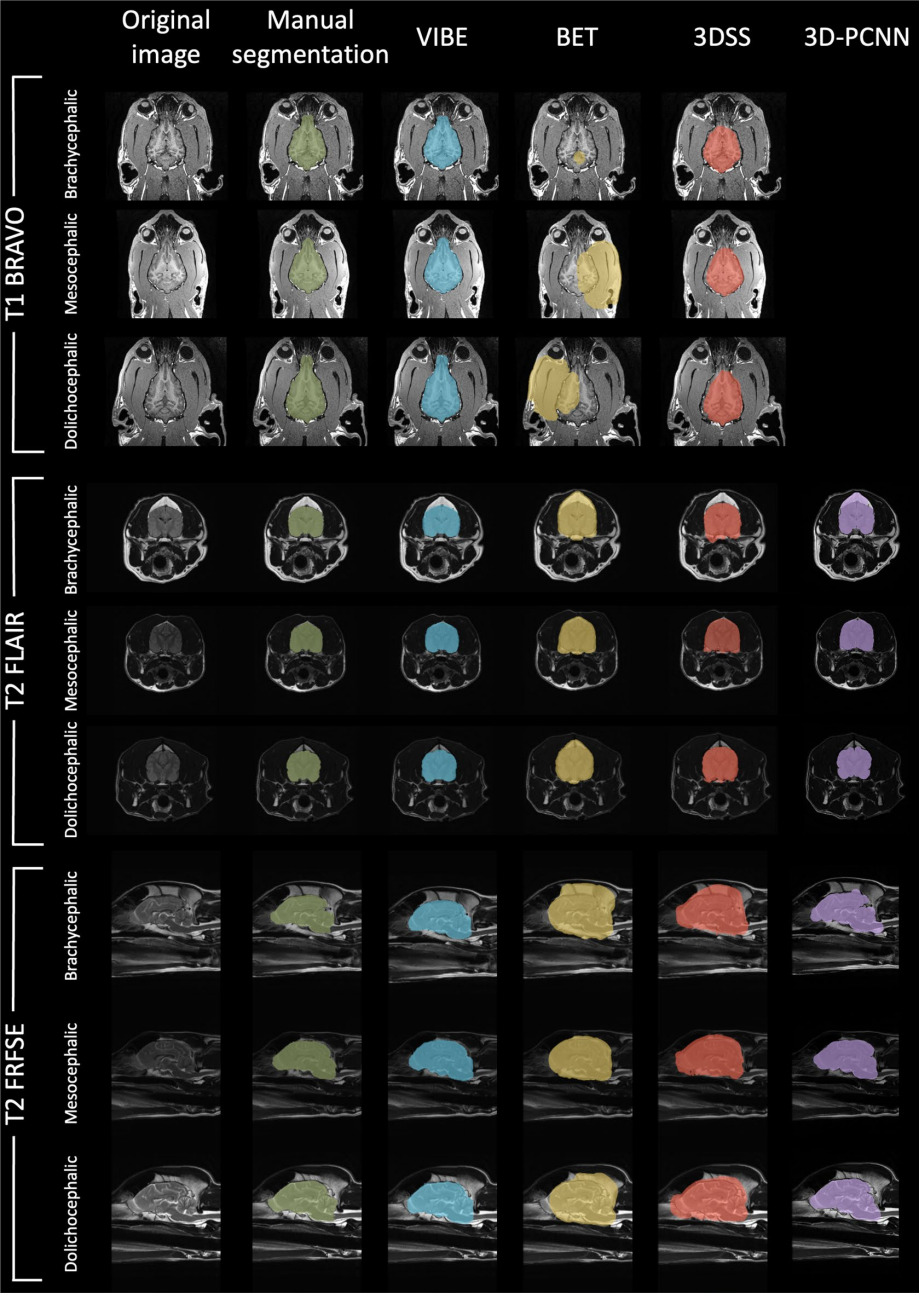
Dogs cohort: comparison of the brain extraction results for each segmentation technique. From left to right: Original image, Manual segmentation, VIBE, BET, 3DSS, and 3D-PCNN. Different sequences (T1 BRAVO, T2 FLAIR and T2 FRFSE) are shown, as well as a sample of each brain shape (brachycephalic, mesocephalic and dolichocephalic).

**Figure 5:**
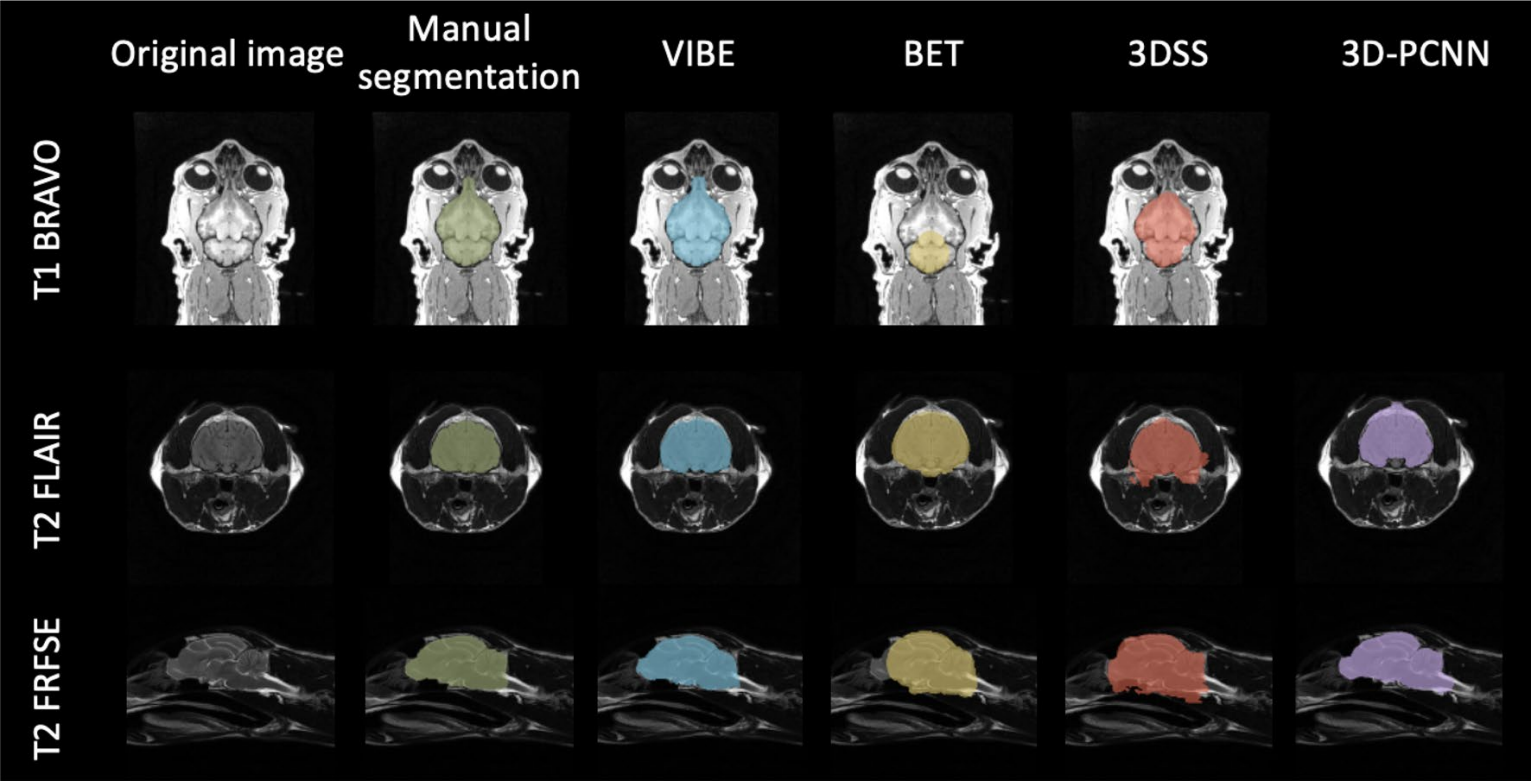
Cats cohort: comparison of the brain extraction results for each segmentation technique. From left to right: Original image, Manual segmentation, VIBE, BET, 3DSS and 3D-PCNN. Different sequences (T1 BRAVO, T2 FLAIR and T2 FRFSE) are shown.

The different sequences have been acquired along the three different anatomical planes: the T1 BRAVO along the dorsal plane, the T2 FRFSE along the sagittal plane, and the T2 FLAIR along the transverse plane. No issues have arisen from the use of different anatomical planes. An example of the results for the different sequences and anatomical planes is visually shown in Fig. 4 (dogs) and in Fig. 5 (cats). The values of Sensitivity and Specificity (see Supplementary Table 1 and Supplementary Fig. 2 for the dogs’ cohort, Supplementary Table 2 and Supplementary Fig. 3 for the cats’ cohort) are both very high overall the considered sequences: $Sensitivity>0.93$ for dogs and $Sensitivity>0.90$ for cats, $Specificity>0.98$ for both dogs and cats. This means that VIBE does not have a defined tendency to either overestimate or underestimate the brain region.

#### Tests on different animals and cranial conformations

**Figure 6:**
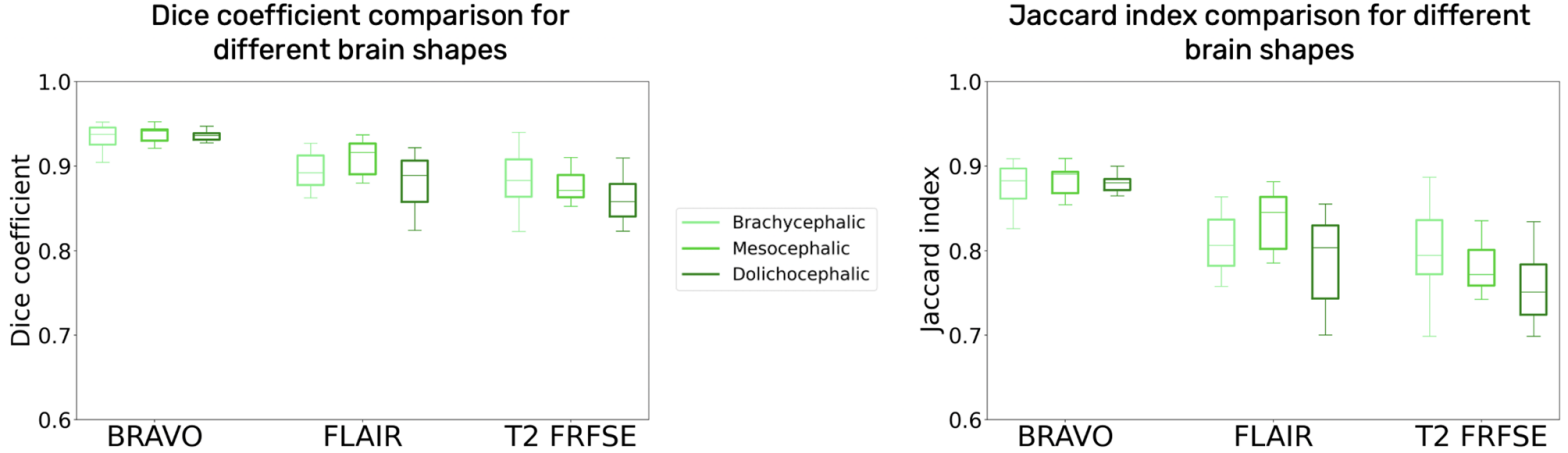
Dice similarity coefficient and Jaccard index Boxplot for the dogs cohort, comparing VIBE results for different cranial conformations (brachycephalic, mesocephalic, dolichocephalic), for three different sequences: T1 BRAVO, T2 FRFSE and T2 FLAIR.

The results for both the dogs cohort and the cats cohort are very similar in terms of Dice Similarity Coefficient and Jaccard index, and it is the case for all the MRI sequences tested. We also tested different cranial conformations in the dogs cohort, which was chosen to contain 10 dogs for each of the cranial shapes: brachycephalic, mesocephalic and dolichocephalic. A brachycephalic dog’s brain might be harder to precisely extract, due to higher differences with respect to the other cranial conformations especially in the rostral portion of the brain, where the olfactory bulb is located. For this reason, the chosen atlas excludes brachycephalic dogs. Nonetheless, there is no significant difference in the quality of the results for different canine cranial conformations. The results of VIBE on the dogs cohort brain extraction, sorted by cranial shape, are reported in Table 5 and Supplementary Table 3, and graphically shown with boxplots in Figure 6 and Supplementary Fig. 4. VIBE is thus adapted for brain extraction of every kind of canine breed and cranial conformation.

**Table 5:** Dogs cohort results of VIBE, sorted by cranial conformations (brachycephalic, mesocephalic, dolichocephalic).

Dogs	T1 BRAVO		T2 FLAIR		T2 FRFSE	
	Dice	Jaccard	Dice	Jaccard	Dice	Jaccard
Brachycephalic	$0.92 \pm 0.02$	$0.86 \pm 0.04$	$0.89 \pm 0.02$	$0.80 \pm 0.03$	$0.88 \pm 0.03$	$0.79 \pm 0.05$
Mesocephalic	$0.93 \pm 0.01$	$0.88 \pm 0.01$	$0.90 \pm 0.04$	$0.82 \pm 0.07$	$0.88 \pm 0.02$	$0.78 \pm 0.04$
Dolicocephalic	$0.936 \pm 0.007$	$0.88 \pm 0.01$	$0.88 \pm 0.03$	$0.79 \pm 0.04$	$0.86 \pm 0.02$	$0.75 \pm 0.04$

Comparison of the mean and standard deviation results of both the Dice similarity coefficient and Jaccard index for three different sequences (T1 BRAVO, T2 FLAIR and T2 FRFSE).

#### Comparison with other algorithms

VIBE outperformed BET and 3DSS for all of the selected contrasts, and for both dogs and cats (see Table 3, 4, Fig. 2, 3, Supplementary Tables 1, 2 and Supplementary Fig. 1, 2). Even after a careful optimisation of their parameters, BET and 3DSS show their limitations to adapt to non-human brains. Canine and feline brain contours are harder to separate from surrounding tissues using classic deformable models. As can be seen in Fig. 4 for dogs, and Fig. 5 for cats, BET and 3DSS often include portions of non-brain tissues connected to the brain, while VIBE, being atlas-based, successfully segments the brain region only. 3D-PCNN, an algorithm originally designed for optimal performance on rodent brains, has the possibility to adapt to other species thanks to its tuning flexibility of the brain size. Its performance in terms of DSC and Jaccard index is slightly inferior to VIBE for the dogs cohort (see Table 3 and Fig. 2), and comparable in quality for the cats cohort (see Table 4 and Fig. 3). Unfortunately, we were not able to optimize 3D-PCNN parameters to use it for brain extraction on T1 BRAVO images: none of the tested parameters produced a mask containing the brain neither on dogs, nor on cats. This limitation is mentioned in the original paper^4^, and is due to lower performance when the contrast between cortex and skull is low.

### Tissues segmentation

**Figure 7:**
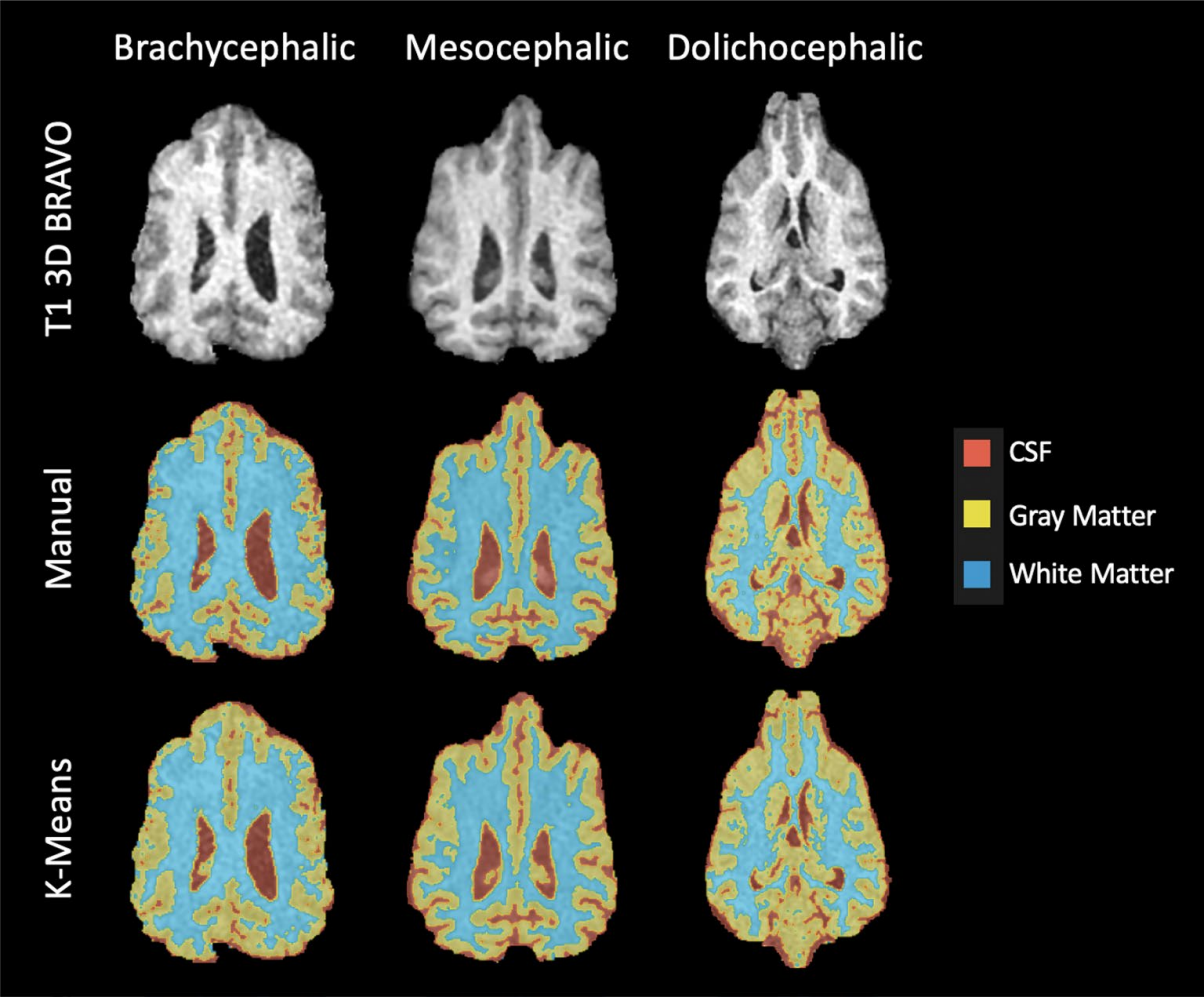
Segmentation of the white matter, gray matter and cerebrospinal fluid (CSF), on three dogs of different cranial conformation (brachycephalic, mesocephalic, dolicocephalic). The acquired T1 3D BRAVO image is compared with manual segmentation and automatic segmentation using *K*-Means clustering.

Brain extraction is a preliminary step that proves to be necessary in many applications. We take the example of brain tissues segmentation, which is useful for quantitative diagnosis and characterization of brain abnormalities^46^. We show how easy it becomes to segment white matter, gray matter and cerebrospinal fluid after using VIBE. Segmenting the brain tissues on a full encephalic MR image can be a difficult task: methods based on the clustering of pixels intensities are biased by the surrounding non-brain tissues. After brain extraction, the classification of brain tissues becomes easier, faster and more accurate. To show this, we picked the 3D T1-weighted BRAVO acquisitions of three different dogs from our cohort: a brachycephalic, a mesocephalic and a dolichocephalic dog (subjects number 1, 13, 25 respectively, see Table 1). For each of them, we chose a dorsal slice in the central part of the brain, where the three brain tissues were well visible. First, we applied the VIBE algorithm to perform brain extraction. Afterwards, a simple *K*-Means^47^ clustering algorithm was applied to perform automatic segmentation of the brain tissues (Fig. 7). $K=3$ was chosen to represent the three tissue classes. Manual segmentation of the brain tissues was also performed on the same slices by an experienced user and verified by veterinary experts, in order to provide a ground truth control. The detailed results of the Dice similarity coefficients and Jaccard indexes, comparing the automatic and manual segmentations, are reported in Table 6. For the three tissues the Dice coefficients span between 0.78 and 0.92, and the Jaccard indexes between 0.64 and 0.86. The lower Dice and Jaccard values are associated to partial volume effects: the pixels affected by partial volume can be evaluated differently by a veterinary expert with respect to the intensity considered by the clustering algorithm. Apart from this exception, occurring in particular on the cerebrospinal fluid segmentation of the mesocephalic dog, the quality of the K-means segmentation is very high. This shows that, after brain extraction, even a very simple yet well established *K*-means clustering can be used to perform high-quality segmentation of brain tissues.

**Table 6:** Brain tissues segmentation performed by *K*-Means clustering on brains skull-stripped by VIBE.

Dogs	White Matter		Gray Matter		Cerebrospinal Fluid	
	Dice	Jaccard	Dice	Jaccard	Dice	Jaccard
Brachycephalic	0.92	0.86	0.84	0.73	0.89	0.81
Mesocephalic	0.89	0.80	0.86	0.75	0.78	0.64
Dolicocephalic	0.91	0.84	0.86	0.75	0.89	0.81

Three dogs with three different cranial conformations (brachycephalic, mesocephalic, dolichocephalic) are reported.

## Discussion

In this paper we presented an automatic brain extraction algorithm, VIBE, that is adapted to veterinary applications. Brain extraction is an important pre-processing algorithm in neuroimaging applications, but we found that popular available tools, such as BET or 3DSS, are not adapted to the veterinary world, being optimised on the human brain. We also tested 3D-PCNN, a tool designed for rodents, that has a greater capability to adapt to different brain sizes and morphologies. It indeed performed well on T2 FRFSE and T2 FLAIR sequences, but it is not optimized for T1 contrast, and thus failed on T1 3D BRAVO sequences. At the best of our knowledge, no unified brain extraction tool exists that can be easily adapted to multiple species. For some species in particular, such as dogs, brain extraction is still either performed manually^48,49^, or in a semi-automatic way. BET is used as preliminary step, then the resulting masks are manually corrected by an expert^50,51^. Recent studies still claim that no trustworthy brain extraction tool exists for dogs, and manual or semi-automatic segmentation is still the only reliable and accurate existing strategy^18^. The choice to use atlas-based brain extraction for VIBE has the advantage to adapt automatically to different animal species, without modifications in its parameters, provided that an atlas exists. It also has the advantage to adapt to different contrasts, since it’s not only a contrast-driven technique: using the atlas as a template of the brain mask makes the adaptation to the brain shape of a new animal easier, with less risks of including surrounding non-brain tissues. For these reasons, the tests we performed considering multiple different variables were all successful with VIBE. We tested VIBE on dogs and cats, the most common animals to be found in veterinary MRI studies, and VIBE was equally efficient for both species. We also checked the segmentation quality depending on different canine brain conformations, and the results showed no significant performance difference between them. In order to adapt to different MRI protocols and applications, we tested VIBE on multiple MRI sequences (T1 BRAVO, T2 FRFSE and T2 FLAIR). The T1 BRAVO gave the best results, due to the fact that it is the same sequence used to build up the atlas. Even if the T2 and T2 FLAIR had a different contrast and were 2D acquisitions, thus a high resampling of the atlas was needed for the segmentation, the results gave a high metrics quality, making VIBE efficient for multiple MRI contrasts. VIBE is also independent on the orientation of the acquisition plane: each of the selected sequences was acquired along one of the three anatomical planes (sagittal, dorsal, transverse), and in all these cases the algorithm was capable to correctly detect the plane and adapt the pre-processing rotations and translations of the atlas accordingly. The last parameter that we considered was the computation time of the two best-performing techniques, 3D-PCNN and VIBE. The average computation time for a T2 FRFSE or FLAIR sequence using VIBE was in average 15 s on a MacBook Pro, while it took an average of 230 s to perform the same brain extraction with 3D-PCNN on a more powerful workstation. On top of the adaptability to multiple contrasts, VIBE has thus also a great advantage in terms of computation time with respect to 3D-PCNN. A high computation time is a limiting factor in the parameters’ optimisation phase, as well as in the application phase, especially for big cohorts. We conclude that VIBE outperforms all the other techniques tested in this paper, and we predict that for every other animal species for which an atlas is available, the VIBE pipeline can be successfully applied. We plan to use VIBE to broaden the tools available in the veterinary neuroimaging field, and in particular in the development pipelines of brain lesions automatic segmentation and characterisation algorithms, to push future research and clinical applications in this field further.

## Supplementary Information

Supplementary Information.

## Data Availability

The datasets analysed during the current study are available from the corresponding author on reasonable request.
