# Supplementary material for: Automatic brain extraction and brain tissues segmentation on multi-contrast animal MRI
Source: Sci Rep. 2023 Apr 19;13:6416. doi: 10.1038/s41598-023-33289-7 (PMC10115851; doi:10.1038/s41598-023-33289-7)
Supplement: Supplementary file 1 — Supplementary Information. [file 41598_2023_33289_MOESM1_ESM.pdf]

Supplementary Data

Automatic brain extraction and brain tissues segmentation on multi-contrast animal MRI

Jamil Nour Eddin[1], Hugo Dorez[1], and Valentina Curcio[1,*]

[1]HawkCell, Marcy-l'Étoile, 69280, France
[*]valentina.curcio@hawkcell.com

Supplementary Tables

Dogs	T1 BRAVO		T2 FLAIR		T2 FRFSE	
	Sensitivity	Specificity	Sensitivity	Specificity	Sensitivity	Specificity
VIBE	0.95 ± 0.02	0.995 ± 0.003	0.93 ± 0.05	0.993 ± 0.004	0.97 ± 0.01	0.984 ± 0.005
BET	0.4 ± 0.3	0.95 ± 0.04	0.989 ± 0.007	0.97 ± 0.01	0.993 ± 0.005	0.95 ± 0.01
3DSS	0.4 ± 0.3	0.98 ± 0.01	0.6 ± 0.3	0.97 ± 0.01	0.70 ± 0.24	0.94 ± 0.08
3D-PCNN	/	/	0.8 ± 0.1	0.995 ± 0.004	0.89 ± 0.07	0.98 ± 0.01

Supplementary Table 1. Dogs cohort results. Comparison of the mean and standard deviation results of both the Sensitivity and Specificity metrics for three different MRI contrasts (T1 BRAVO, T2 FLAIR and T2 FRFSE) and different algorithms (VIBE, BET, 3DDS and 3D-PCNN).

Cats	T1 BRAVO		T2 FLAIR		T2 FRFSE	
	Sensitivity	Specificity	Sensitivity	Specificity	Sensitivity	Specificity
VIBE	0.93 ± 0.04	0.9981 ± 0.0009	0.91 ± 0.04	0.997 ± 0.001	0.90 ± 0.02	0.995 ± 0.002
BET	0.7 ± 0.4	0.97 ± 0.03	0.94 ± 0.08	0.96 ± 0.03	0.80 ± 0.13	0.90 ± 0.07
3DSS	0.7 ± 0.3	0.99 ± 0.01	0.66 ± 0.41	0.89 ± 0.08	0.74 ± 0.29	0.90 ± 0.07
3D-PCNN	/	/	0.90 ± 0.05	0.997 ± 0.001	0.90 ± 0.02	0.995 ± 0.003

Supplementary Table 2. Cats cohort results. Comparison of the mean and standard deviation results of both the Sensitivity and Specificity metrics for three different MRI contrasts (T1 BRAVO, T2 FLAIR and T2 FRFSE) and different algorithms (VIBE, BET, 3DDS and 3D-PCNN).

Dogs	T1 BRAVO		T2 FLAIR		T2 FRFSE	
	Sensitivity	Specificity	Sensitivity	Specificity	Sensitivity	Specificity
Brachycephalic	0.96 ± 0.01	0.995 ± 0.003	0.95 ± 0.02	0.992 ± 0.003	0.97 ± 0.01	0.985 ± 0.004
Mesocephalic	0.95 ± 0.03	0.99 ± 0.33	0.93 ± 0.05	0.993 ± 0.004	0.97 ± 0.01	0.987 ± 0.004
Dolicocephalic	0.94 ± 0.03	0.995 ± 0.004	0.90 ± 0.05	0.994 ± 0.005	0.981 ± 0.006	0.982 ± 0.006

Supplementary Table 3. Dogs cohort results of VIBE, sorted by cranial conformations (brachycephalic, mesocephalic, dolichocephalic). Comparison of the mean and standard deviation results of both the Sensitivity and Specificity for three different sequences (T1 BRAVO, T2 FLAIR and T2 FRFSE).

Supplementary Figures

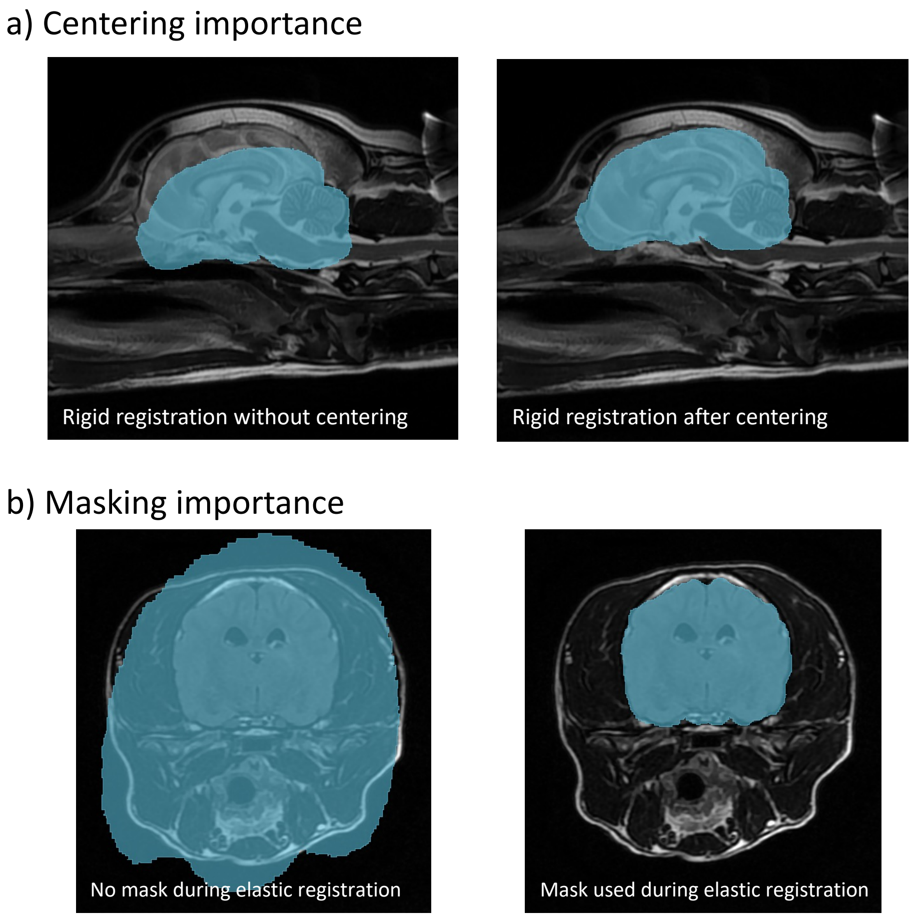

Supplementary Figure 1. a) The centering step done through the Felsenszwalb's segmentation for brain detection is key for a good rigid registration output. Model-to-image registration fails without proper alignment initialization, as shown in the left image, where no centering initialisation was performed, with respect to the right image, where centering initialisation was performed. b) The use of a mask that identifies roughly the brain region is fundamental in the elastic registration step. Being it a model-to-image registration, the atlas risks to get completely deformed if it is free to span the whole image range.

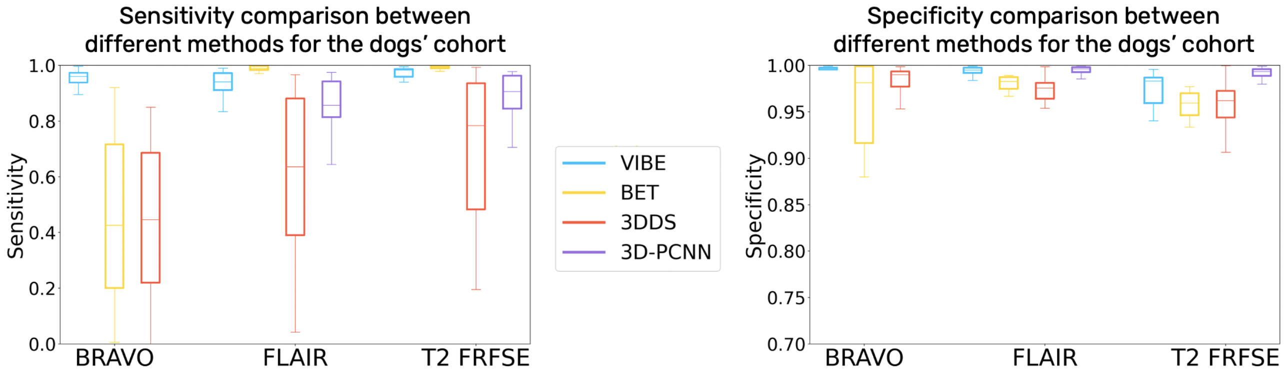

Supplementary Figure 2. Sensitivity and specificity boxplot for the dogs cohort, comparing VIBE, BET, 3DSS and 3D-PCNN for three different sequences: T1 BRAVO, T2 FLAIR and T2 FRFSE.

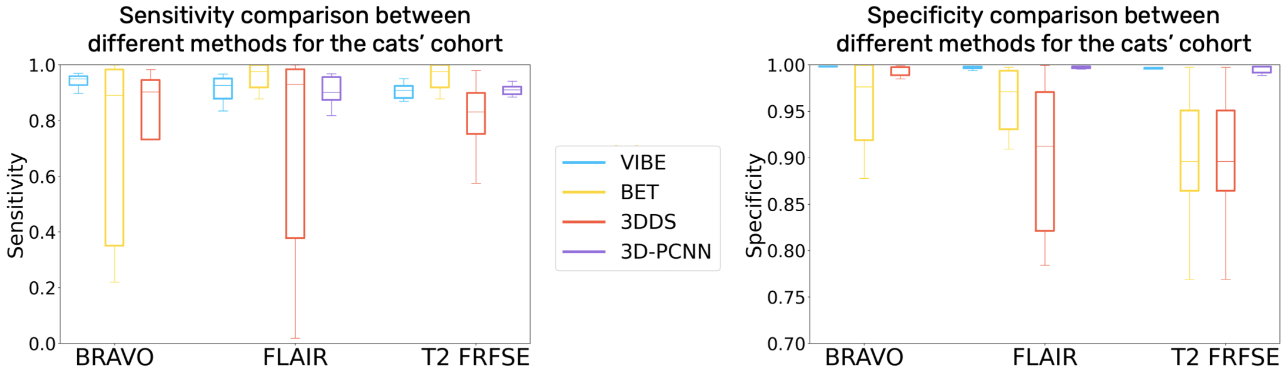

Supplementary Figure 3. Sensitivity and specificity boxplot for the cats cohort, comparing VIBE, BET, 3DSS and 3D-PCNN for three different sequences: T1 BRAVO, T2 FLAIR and T2 FRFSE.

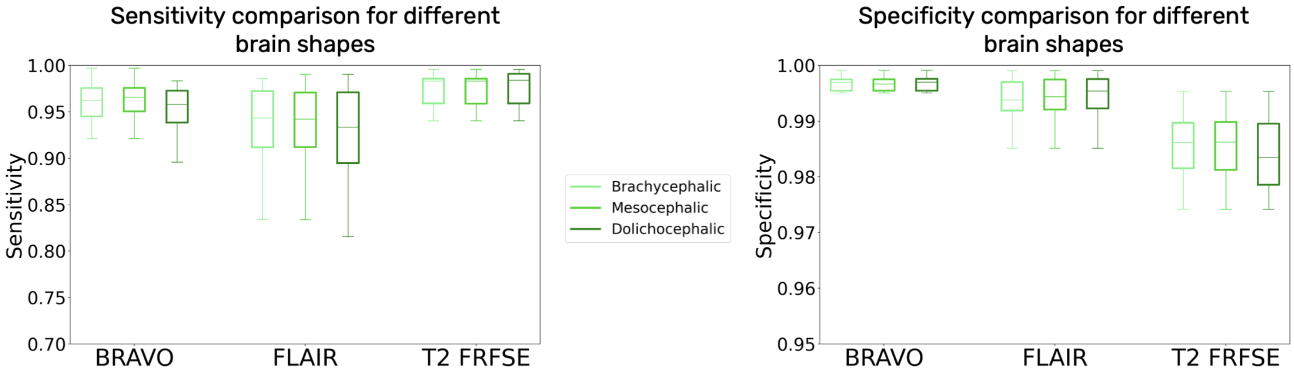

Supplementary Figure 4. Sensitivity and Specificity boxplot for the dogs cohort, comparing VIBE results for different cranial conformations (brachycephalic, mesocephalic, dolichocephalic), for three different sequences: T1 BRAVO, T2 FRFSE and T2 FLAIR.
